# Tween 40 pretreatment of unwashed water-insoluble solids of reed straw and corn stover pretreated with liquid hot water to obtain high concentrations of bioethanol

**DOI:** 10.1186/1754-6834-6-159

**Published:** 2013-11-09

**Authors:** Jie Lu, Xuezhi Li, Ruifeng Yang, Jian Zhao, Yinbo Qu

**Affiliations:** 1 State Key Laboratory of Microbial Technology, Shandong University, Jinan City, Shandong Province 250100, China; 2 Dalian Polytechnic University, Dalian 116034, China

**Keywords:** Tween 40 pretreatment, Liquid hot water pretreatment, Biofuel, Bioethanol, Corn stover, Reed straw, Semi-simultaneous saccharification and fermentation

## Abstract

**Background:**

Liquid hot water (LHW) pretreatment is an effective and environmentally friendly method to produce bioethanol with lignocellulosic materials. In our previous study, high ethanol concentration and ethanol yield were obtained from water-insoluble solids (WIS) of reed straw and corn stover pretreated with LHW by using fed-batch semi-simultaneous saccharification and fermentation (S-SSF). However, high cellulase loading and the large amount of wash water possibly limit the practical application of LHW pretreatment. To decrease cellulase loading and the amount of wash water, we performed Tween 40 pretreatment before WIS was subjected to bioethanol fermentation.

**Results:**

Results showed that the optimum conditions of Tween 40 pretreatment were as follows: Tween 40 concentration of 1.5%, WIS-to-Tween 40 ratio of 1:10 (w/v), and pretreatment time of 1 hour at ambient temperature. After Tween 40 pretreatment, cellulase loading could be greatly reduced. After Tween 40 pretreatment, the residual liquid could be recycled for utilization but slightly affected ethanol concentration and yield. The unwashed WIS could obtain a high ethanol concentration of 56.28 g/L (reed straw) and 52.26 g/L (corn stover) by Tween 40 pretreatment using fed-batch S-SSF. Ethanol yield reached a maximum of 69.1% (reed straw) and 71.1% (corn stover).

**Conclusions:**

Tween 40 pretreatment was a very effective and less costly method with unwashed WIS. This pretreatment could greatly reduce cellulase loading and save wash water. Higher ethanol concentration was obtained almost without reducing ethanol yield.

## Background

Bioethanol has been widely used as a substitute for fossil fuels [1]. The use of bioethanol produced from lignocellulosic material can reduce the dependence on fossil fuels [2]. In general, lignocellulosic materials are subjected to bioethanol conversion performed in three steps: pretreatment; enzymatic hydrolysis; and fermentation [1]. Pretreatment is crucial to determine conversion efficiency. Studies have investigated and proposed many pretreatment materials/methods, such as alkaline [3], steam explosion [4], ammonia fiber expansion [5], organic solvent [6], and diluted acid [7]. One of the most promising pretreatment processes of lignocelluloses material is liquid hot water (LHW) pretreatment. LHW pretreatment has been considered as an environmentally friendly technology. LHW has been shown to remove most of the hemicelluloses, but a large amount of lignin is retained in water-insoluble solids (WIS). Cellulase can be adsorbed on lignin surfaces during enzymatic hydrolysis, thereby deactivating cellulase. Studies have indicated that surfactant additives can improve enzymatic hydrolysis and bioethanol fermentation of lignocellulosic biomass [8-12]. Tween additives can effectively improve cellulase efficiency during enzymatic hydrolysis and fermentation of lignocellulosic materials [13,14]. For example, the simultaneous saccharification and fermentation (SSF) of steam-pretreated softwood was improved by the addition of Tween 20 due to a combination of increased hydrolysis rate and improved yeast fermentation [15]. Research about the effects of surfactant on SSF of steam-exploded poplar has also shown that the ethanol yield could be increased by 6% by the addition of Tween 80 [16]. Ooshima *et al*. reported that the rate of SSF of pure cellulose (Avicel) was slightly enhanced by adding Tween 20 [17]. Studies about the mechanism of Tween additives on enzymatic hydrolysis and fermentation of lignocellulosic materials have also been proposed [18-21]. The mechanism mainly focuses on three aspects: protecting free cellulase from deactivation; decreasing cellulase protein adsorption on the substrate; and reducing unproductive binding of enzymes to lignin. Tween additives contain hydrophilic ethylene glycol head groups and a hydrophobic alkyl tail. Absorption of the hydrophobic alkyl group to a hydrophobic surface exposes the hydrophilic ethylene glycol chains, thus making the surface resistant to non-specific protein adsorption [22]. The commonly used Tween additives include Tween 20, Tween 40, Tween 60, and Tween 80. These additives contain the same hydrophilic head group with different lengths of hydrophobic alkyl tail. Different methods of applying Tween additives produce different results of bioethanol fermentation. Tween additives can be applied mainly in three stages: during the pretreatment process [23]; in the enzymatic hydrolysis stage [24]; and in the fermentation stage [24]. However, few reports are available on Tween 40 pretreatment prior to the fed-batch semi-simultaneous saccharification and fermentation (S-SSF) of lignocellulosic biomass. The present study aimed to confirm the effect of Tween 40 pretreatment on the fermentation digestibility of unwashed WIS of reed straw and corn stover pretreated with LHW. Several pretreatment methods were compared. The positive effect of Tween 40 pretreatment with unwashed WIS was observed. The effect of Tween 40 was possibly related to process conditions, so we also investigated the process conditions of Tween 40 pretreatment, such as temperature, time, concentration, and ratio of WIS-to-Tween 40. The residual liquid of Tween 40 pretreatment was recycled to save wash water. Cellulase dosage and feeding methods in fed-batch S-SSF process were also researched to obtain high ethanol concentration. This article presents the results.

## Results and discussion

### Effect of different pretreatment methods of Tween 40 on ethanol concentration using unwashed and washed WIS as lignocellulosic substrates

**Figure 1:**
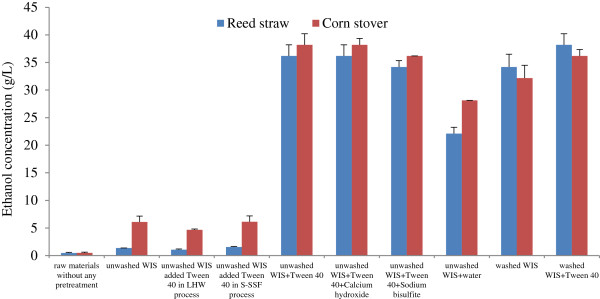
**Effect of different pretreatment methods with unwashed and washed WIS on ethanol concentration.** The conditions of Tween 40 pretreatment were as follows: Tween 40 concentration, 1%; the ratio of WIS-to-Tween 40, 1:10 (w/v); pretreatment temperature, 50°C; and pretreatment time, 1 hour. The fed-batch S-SSF conditions were as follows: 1 g dry weight biomass; cellulase loading, 20 FPU/g oven-dried WIS; pre-hydrolysis temperature, 50°C; pre-hydrolysis time, 18 hours; fermentation temperature, 36°C; and fermentation time, 72 hours, and after 6 hours of pre-hydrolysis, 1 g dry weight biomass was supplemented into the flask.

Tween 40 was used as an additive in different processes, for example, LHW pretreatment, S-SSF, or as a single pretreatment stage to treat WIS in this study, and the effect of different pretreatment methods of Tween 40 on ethanol concentration using unwashed and washed WIS as substrates is shown in Figure 1. Using untreated raw materials as substrates, the ethanol concentration after fed-batch S-SSF was very low (0.47 g/L for reed straw and 0.48 g/L for corn stover). Therefore, the raw materials without any pretreatment were not suitable for the production of ethanol. The ethanol concentrations of unwashed WIS without any pretreatment were 1.36 g/L (reed straw) and 6.09 g/L (corn stover) (Figure 1). Ethanol concentration was also very low, indicating that unwashed WIS was not suitable for direct fermentation and should undergo pretreatment for follow-up fermentation. The unwashed WIS was washed with water until a neutral condition was obtained. Ethanol concentration significantly increased and reached 34.17 g/L (reed straw) and 32.16 g/L (corn stover) when the washed WIS was used for fermentation. However, a large amount of wash water was used in the washing process, thereby increasing production cost. The ethanol concentration was approximately equal to that of the washed WIS when the unwashed WIS was pretreated with Tween 40, but the washed WIS was treated with Tween 40 to slightly increase ethanol concentration compared with washed WIS without Tween 40, indicating that Tween 40 pretreatment is more suitable for unwashed WIS. To confirm the effect of Tween 40, we used water as a control treatment instead of Tween 40 in the experiment. Ethanol concentrations obtained from water-pretreated unwashed WIS were 22.11 g/L (reed straw) and 28.14 g/L (corn stover), respectively. This result was lower than that obtained by Tween 40 pretreated unwashed WIS (36.18 g/L for reed straw and 38.19 g/L for corn stover, respectively), indicating that Tween 40 pretreatment positively affects the S-SSF ability of the unwashed WIS. Some studies have indicated that calcium hydroxide and sodium bisulfite can eliminate the negative effect of inhibitors because unwashed WIS contains a large number of inhibitors of yeast [25]. Calcium hydroxide and sodium bisulfite added to Tween 40 pretreatment stage was performed. Figure 1 also shows that the ethanol concentrations were retained (calcium hydroxide) or reduced (sodium bisulfite) when the two chemicals were added in the S-SSF of unwashed WIS. Therefore, Tween 40 is possibly the most suitable pretreatment method for unwashed WIS, and detoxication treatment of unwashed WIS for eliminating the negative effect of inhibitors produced by LHW pretreatment is not required.

The Tween 40 was also added into the LHW and S-SSF process to evaluate its function on improving ethanol production, since they could shorten the whole product process and save energy. However, the results show that both ethanol concentrations did not increase (Figure 1). This indicated that adding Tween 40 to either the LHW or S-SSF process was not suitable for unwashed WIS.

Many studies have reported the effect of the surfactant Tween series on enzymatic hydrolysis and SSF and its mechanism [12,15,18-21]. The studies showed that the addition of surfactant Tween improved enzymatic hydrolysis yields and ethanol production. Surfactants can enhance enzymatic digestibility by: 1) changing the substrate structure to make it more accessible to enzymes; 2) stabilizing enzymes to prevent denaturation; 3) increasing positive interactions between substrates and enzymes; and 4) reducing enzyme non-productive binding to lignin and other molecules involved in cellulase activity [24]. Tween contains hydrophilic ethylene glycol head groups and a hydrophobic alkyl tail. Hydrophilic surfactants have been reported to be useful in extracting hydrophobic degradation products from lignin and hemicellulose. Based on the mechanisms, pretreatment with Tween 40 possibly removed some degradation products from lignin and hemicellulose, which were contained in unwashed WIS and had a negative effect on enzymatic hydrolysis and fermentation. Besides, the Tween 40 acted as the above surfactants to enhance enzymatic digestibility of unwashed WIS.

### Optimization of process conditions of Tween 40 pretreatment

#### Effect of Tween 40 pretreatment temperature on ethanol concentration

**Figure 2:**
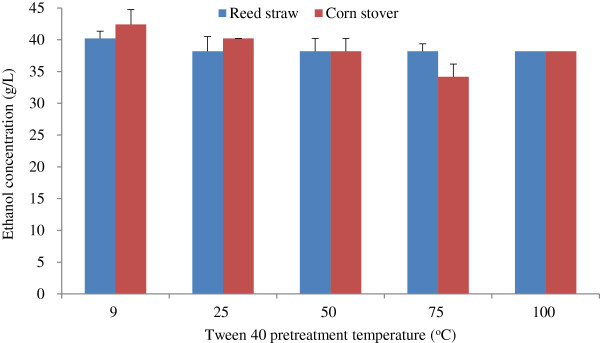
**Effect of Tween 40 pretreatment temperature on ethanol concentration.** The conditions of Tween 40 pretreatment and fed-batch S-SSF were the same as that in Figure 1, except pretreatment temperature. S-SSF, semi-simultaneous saccharification and fermentation.

The effect of Tween 40 pretreatment temperature on ethanol concentration is shown in Figure 2. The ethanol concentrations from reed straw and corn stover varied. The ethanol concentration obtained from reed straw remained almost unchanged as Tween 40 pretreatment temperature increased. By contrast, ethanol concentration obtained from corn stover decreased slightly as Tween 40 pretreatment temperature increased before 100°C. Taking supplied water temperature in winter into consideration, we conducted the Tween 40 pretreatment at the lowest temperature of 9°C, and showed the pretreatment temperature had little effect on ethanol yield. Thus, the proposed Tween 40 pretreatment may be conducted at room temperature.

#### Effect of Tween 40 pretreatment time on ethanol concentration

**Figure 3:**
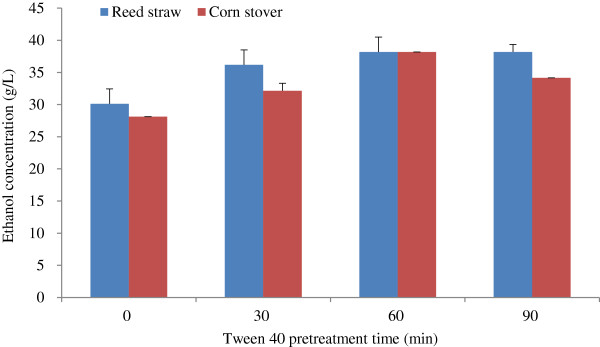
**Effect of Tween 40 pretreatment time on ethanol concentration.** The conditions of Tween 40 pretreatment and fed-batch S-SSF were the same as that in Figure 1, except Tween 40 pretreatment temperature of 25°C. S-SSF, semi-simultaneous saccharification and fermentation.

The effect of Tween 40 pretreatment time on ethanol concentration is shown in Figure 3. Tween 40 pretreatment time ranged from 0 to 90 minutes. At 0 minutes, the unwashed WIS was washed directly with Tween 40 solution at 25°C. Figure 3 shows that the change rule of the ethanol concentrations obtained from reed straw and corn stover were similar. Ethanol concentration increased as Tween 40 pretreatment time was prolonged from 0 to 60 minutes. However, for reed straw, ethanol concentration neither increased nor decreased when pretreatment time was further prolonged, and for corn stover, ethanol concentration decreased slightly. Therefore, the suitable Tween 40 pretreatment time was 60 minutes.

#### Effect of Tween 40 concentration on ethanol concentration

**Figure 4:**
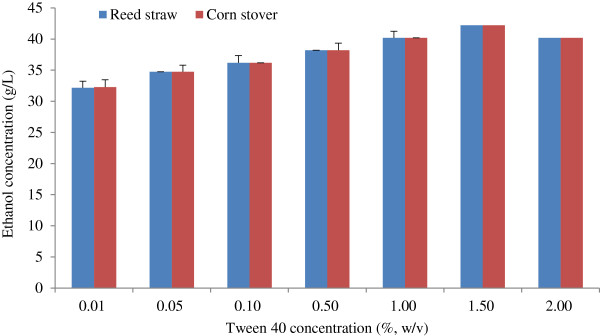
**Effect of Tween 40 concentration on ethanol concentration.** The conditions of Tween 40 pretreatment and fed-batch S-SSF were the same as that in Figure 3. S-SSF, semi-simultaneous saccharification and fermentation.

The effect of Tween 40 concentration on ethanol concentration is shown in Figure 4, in which the ethanol concentration was almost the same for the reed straw and corn stover pretreated at different Tween 40 concentrations. Ethanol concentration initially increased and then decreased as Tween 40 concentration increased. The highest ethanol concentration of 42.21 g/L for the reed straw and corn stover was observed at Tween 40 concentration of 1.5%. Thus, Tween 40 concentration of 1.5% was used for the subsequent experiments.

#### Effect of WIS-to-Tween 40 ratio on ethanol concentration

**Figure 5:**
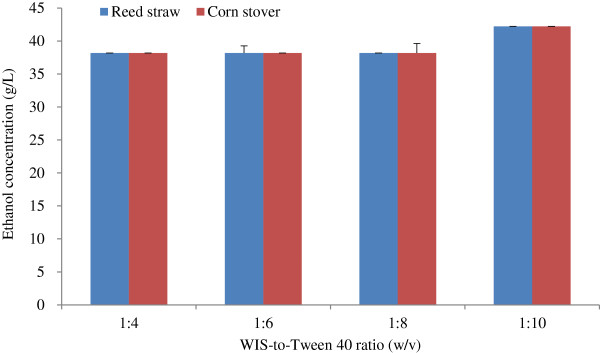
**Effect of WIS-to-Tween 40 ratio on ethanol concentration.** Tween 40 concentration of 1.5% was used in Tween 40 pretreatment, and other conditions in Tween 40 pretreatment and fed-batch S-SSF were the same as that in Figure 4. S-SSF, semi-simultaneous saccharification and fermentation.

The effect of WIS-to-Tween 40 ratio (w/v) on ethanol concentration is shown in Figure 5, wherein the ethanol concentration did not increase when the WIS-to-Tween 40 ratio ranged from 1:4 to 1:8. However, the ethanol concentration increased when the WIS-to-Tween 40 ratio increased to 1:10. More Tween 40 will be consumed when the ratio of WIS to Tween increased. Thus, the WIS-to-Tween 40 of 1:10 was appropriate for ethanol production.

#### Effects of the recycling frequency of residual liquid after Tween 40 pretreatment on ethanol concentration

**Figure 6:**
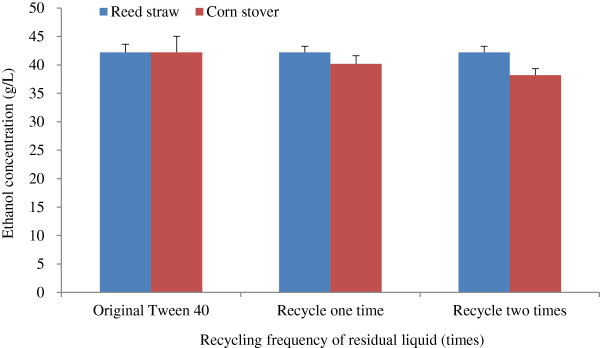
**Effects of recycling frequency of residual liquid after Tween 40 pretreatment on ethanol concentration.** Cellulase loading of 25 FPU/g oven-dried WIS was used in fed-batch S-SSF process, and other conditions were the same as that in Figure 5. S-SSF, semi-simultaneous saccharification and fermentation; WIS, water-insoluble solids.

The effects of recycling frequency of residual liquid after Tween 40 pretreatment on ethanol concentration are shown in Figure 6, in which reed straw was slightly different from corn stover. Ethanol concentration obtained from reed straw remained almost unchanged, whereas ethanol concentration obtained from corn stover decreased slightly as the Tween 40 recycle frequency increased. Overall, the pretreatment residual liquid may be recycled to reduce Tween 40 consumption and decrease the cost of the Tween 40 pretreatment. An optimum number of recycling of the residual liquid will be investigated in further work.

In summary, to obtain a high ethanol yield, WIS obtained from the LHW pretreatment process generally needs to be washed with water or detoxicated with chemicals such as calcium hydroxide to remove some inhibitors that have a negative effect on yeast. A large amount of wash water will be consumed in the washing process, which could lead to an increase in process cost and environmental pollution. For example, approximately 200 mL of water was used to wash 3 g WIS until the pH reached 7, which means that approximately 67 m^3^ of wash water per ton dry weight of unwashed WIS needs to be consumed. However, as a substitution for washing with water, the Tween 40 pretreatment was used in ethanol production from biomass to save large amounts of wash water. The Tween 40 pretreatment could be performed at room temperature, and the residual liquid after Tween 40 pretreatment could also be recycled to save the surfactant dosage and reduce the risk of environmental pollution caused by secondary wastewater generated after Tween 40 pretreatment. Thus, the Tween 40 pretreatment may develop into an energy-saving and environmentally friendly method that could be used in ethanol production from biomass.

### Optimization of process conditions of fed-batch S-SSF

#### Effect of cellulase loading in fed-batch S-SSF on ethanol concentration

**Figure 7:**
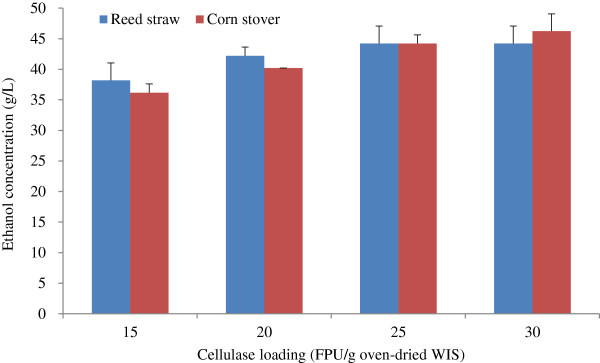
**Effect of cellulase loading on ethanol concentration.** The conditions of Tween 40 pretreatment and fed-batch S-SSF were the same as that in Figure 6. S-SSF, semi-simultaneous saccharification and fermentation.

High production cost is the main obstacle hindering the commercialization of bioethanol. An important and expensive input into the biomass conversion system is enzyme loading, which can amount to approximately 60% of the whole cost [26]. Hence, enzyme dosage should be as low as possible. In this study, the effects of cellulase loadings (presented as FPU/g oven-dried WIS) on the fed-batch S-SSF of unwashed WIS pretreated with Tween 40 were investigated. The ethanol concentrations produced with different cellulase loadings are shown in Figure 7. The ethanol concentration increased when the cellulase loading increased from 15 FPU/g to 25 FPU/g of oven-dried WIS in fed-batch S-SSF (Figure 7). The increase in ethanol concentration was not evident for a higher dosage of cellulase. Therefore, cellulase loading of 25 FPU/g oven-dried WIS was sufficient. In a previous study [27], cellulase loading reached 30 FPU/g to 40 FPU/g of oven-dried WIS (reed straw) and 50 FPU/g of oven-dried WIS (corn stover) when the raw materials were not pretreated with Tween 40 prior to S-SSF. Cellulase loading could be reduced by approximately 40% to 50%. This result occurred possibly because Tween 40 pretreatment decreases adsorption of cellulase to the WIS and cellulase deactivation due to lignin. This phenomenon has potential economic implications because the cost of cellulase is a major contributor to process expenses, considering that the price of Tween 40 is lower than that of cellulase. This work will be continued, with the aim of finding cheaper surfactants with the same positive effects as Tween 40.

#### Effect of fed-batch methods on ethanol concentration and ethanol yield

To enhance ethanol concentration and yield, we performed different fed-batch methods. In a previous study, the fed-batch methods of feeding one time at the pre-hydrolysis time of 6 hours obtained high ethanol concentration and yield [27]. On this basis, WIS was fed twice at the pre-hydrolysis time of 16 hours and fermentation time of 6 hours after feeding time twice, respectively. The ethanol concentration and yield at different feeding methods of fed-batch S-SSF are shown in Table 1. Five fed-batch methods (numbered 1 to 5) were evaluated, and the two materials were then compared (Table 1). The results showed that all of the fed-batch methods were beneficial to ethanol production from reed straw and corn stover. Number 5 fed-batch method could obtain a maximum of 56.28 g/L of ethanol concentration and 69.1% ethanol yield (based on glucan of unwashed WIS from LHW process) with reed straw. Number 3 fed-batch method could obtain a maximum of 52.26 g/L ethanol concentration and 71.1% ethanol yield with corn stover. Considering the yield of WIS in LHW pretreatment, the ethanol yield was also calculated on the basis of dry weight of the original feedstock that enters the process, which was 197 g ethanol per kg of reed straw and 233 g ethanol per kg of corn stover, respectively. In future studies, ethanol concentration and yield could be further improved if the fed-batch methods are systematically optimized.

**Table 1:** Ethanol concentration and yield at different feed methods using fed-batch S-SSF

**Trial number**	**Dry weight (g) of WIS fed at different times**^**b**^			**Volume of fermentation broth (mL) after feeding**^**a**^		**Reed straw**			**Corn stover**		
	**At 6 hours of pre-hydrolysis**	**At 16 hours of pre-hydrolysis**	**At 6 hours of fermentation**	**Reed straw**	**Corn stover**	**Ethanol concentration (g/L)**	**Ethanol yield (%)**	**Ethanol yield (g/kg feedstock)**	**Ethanol concentration (g/L)**	**Ethanol yield (%)**	**Ethanol yield (g/kg feedstock)**
1	1	-	-	11.44	11.60	41.21 ± 1.42	74.4	248	39.20 ± 2.84	70.3	230
2	1	0.5	-	12.16	12.40	46.23 ± 2.84	71.0	237	51.26 ± 1.42	78.6	257
3	1	1	-	12.88	13.20	41.21 ± 1.42	55.9	186	52.26 ± 2.84	71.1	233
4	1	0.5	0.5	12.88	13.20	53.27 ±1.42	72.3	241	47.24 ± 1.42	64.3	211
5	1	1	0.5	13.60	14.00	56.28 ± 0	69.1	197	52.26 ± 0	64.6	212

^a^Calculated value based on water content of fed WIS. The moisture of fed WIS was 59.02% for reed straw and 61.60% for corn stover; ^b^weight of initial WIS was 1 g in the S-SSF. The Tween 40 pretreatment conditions were as follows: Tween 40 concentration, 1.5%; ratio of WIS-to-Tween 40, 1:10 (w/v); pretreatment temperature, 25°C; and pretreatment time, 1 hour. The fed-batch S-SSF conditions were as follows: cellulase loading, 25 FPU/g oven-dried WIS; pre-hydrolysis temperature, 50°C; pre-hydrolysis time, 18 hours; fermentation temperature, 36°C; and fermentation time, 72 hours. S-SSF, semi-simultaneous saccharification and fermentation.

### Comparison of several ethanol productions using Tween as an additive found in the literature and in the current study

The data obtained in this study highlighted the importance of the use of the Tween 40 pretreatment method prior to fed-batch S-SSF to increase ethanol concentration and decrease cellulase loading. Several studies have produced ethanol by using Tween as an additive. Table 2 shows a comparison of several ethanol productions in which Tween was used as an additive. In contrast to the results of other studies, the ethanol concentration in the current study reached 56.28 g/L (reed straw) and 52.26 g/L (corn stover), a significantly higher ethanol concentration compared with other studies. Ethanol yield reached 69.1% (reed straw) and 71.1% (corn stover) at a relatively low dosage of cellulase. One of the advantages of Tween 40 pretreatment prior to S-SSF was that the process was beneficial to unwashed WIS, which contains a large number of yeast inhibitors. The usual approach is to use large amounts of water to wash inhibitors until neutral conditions are achieved for obtaining high ethanol yield from unwashed WIS, which could lead to large amounts of wash water consumption. The Tween 40 pretreatment could be performed at room temperature and atmosphere pressure, and the residue liquid of Tween 40 pretreatment could be recycled, leading to a decrease in process cost. Thus, Tween 40 pretreatment is an environmentally friendly, energy-saving, and low-cost method for ethanol production with unwashed WIS. In our further work, Tween 40 pretreatment techniques used to obtain consistently high ethanol results will be developed to improve process efficiency and decrease pretreatment cost and cellulase loadings in subsequent enzymatic hydrolysis of WIS. The mechanism of Tween 40 pretreatment that could improve the fermentable digestibility of unwashed WIS will also be investigated in detail.

**Table 2:** Comparison of several ethanol productions using Tween as an additive found in the literature and in the current study

**Raw material**	**Pretreated method**	**Tween additives**	**Adding method of Tween**	**Fermentation method**	**Particle size of raw material**	**Washed or unwashed with water**	**Prehydrolysis time (hours) + fermentation time (hours)**	**Enzyme loadings**	**Ethanol concentration(g/L)**	**Ethanol yield (%)**	**Reference**
Reed straw	LHW	Tween 40	Prior to fermentation	Fed-batch S-SSF	3 to 5 cm	Unwashed	18 + 72	25 FPU cellulase/g oven-dried WIS	56.28	69.1	This study
Corn stover	LHW	Tween 40	Prior to fermentation	Fed-batch S-SSF	4 to 7 cm	Unwashed	18 + 72	25 FPU cellulase/g oven-dried WIS	52.26	71.1	This study
Reed straw	LHW	-	-	Fed-batch S-SSF	20 to 80 mesh	Washed	18 + 72	40 FPU cellulase/g oven-dried WIS	39.40	75.1	[27]
Corn stover	LHW	-	-	Fed-batch S-SSF	20 to 80 mesh	Washed	18 + 72	40 FPU cellulase/g oven-dried WIS	39.40	74.4	In press
Wheat straw	Sulfuric acid	Tween 20	In the fermentation stage	SSF	40 mesh	Washed	72	20 FPU cellulase and 40 CBU β-glucosidase/g glucan	12.4	73.9	[24]
Spruce	Steam pretreatment	Tween 20	In the fermentation stage	SSF	2 to 10 mm	Washed	48	44 FPU/g cellulose	20 to 25	92	[15]
Sugarcane bagasse	Dilute ammonia	Tween 80	In the pretreatment stage	SSF	0.05 to 1.5 cm	Washed	72	30 FPU Spezyme CP and 30 CBU Novozyme 188/g glucan	18 g/100 g of dry biomass	69	[23]
Sugarcane bagasse	Dilute ammonia	Tween 20	In the pretreatment stage	SSF	0.05 to 1.5 cm	Washed	72	30 FPU Spezyme CP and 30 CBU Novozyme 188/g glucan	15 g/100 g of dry biomass	59	[23]

CBU, cellobiase unit; LHW, liquid hot water; SSF, simultaneous saccharification and fermentation; S-SSF, semi-simultaneous saccharification and fermentation; WIS, water-insoluble solids.

## Conclusions

Tween 40 pretreatment prior to bioethanol fermentation of unwashed WIS is a very effective and less costly method of ethanol production with unwashed WIS obtained from LHW pretreatment of corn stover and reed straw. This pretreatment could greatly reduce cellulase loading and save wash water. Higher ethanol concentration was obtained almost without decreasing ethanol yield. The optimum conditions of the Tween 40 pretreatment were as follows: Tween 40 concentration of 1.5%, WIS-to-Tween 40 ratio of 1:10 (w/v), and Tween 40 pretreatment time of 1 hour at ambient temperature. After Tween 40 pretreatment, cellulase loading could be greatly reduced. Residual liquid obtained after Tween 40 pretreatment could be recycled. Unwashed WIS could obtain high ethanol concentrations of 56.28 g/L for reed straw and 52.26 g/L for corn stover by conducting Tween 40 pretreatment prior to bioethanol fermentation with fed-batch S-SSF. Ethanol yields were 69.1% for reed straw and 71.1% for corn stover.

## Materials and methods

### Materials

The reed species *Panjin 101* and *Panjin 6* were provided by Yingkou Papermaking Mill, Liaoning Province, China. The reed straw was cut into 3 cm to 5 cm lengths in the mill. Corn stover was collected from a field near Jinzhou New District (Dalian, China). Corn stover was manually cut into pieces of 4 cm to 7 cm in the laboratory. Samples were then homogenized and stored in a plastic bag for subsequent experiments. The chemical compositions of reed straw and corn stover are shown in Table 3. The commercial cellulase used for the fermentation was purchased from Imperial Jade Biotechnology Co, Ltd, Ningxia, China. *Saccharomyces cerevisiae* was purchased from Angel Yeast Co, Ltd, Hubei, China. The trade name of the yeast was Angel Super Alcohol Active Dry Yeast (molasses base). The yeast was activated prior to fermentation. Approximately 1 g of dry yeast was added to 20 mL of 5% sterilized glucose solution, activated at 38°C for 1 hour, cooled to 28°C to 30°C, and used in the fermentation experiment. The yeast features tolerance with acid (pH 2.5) (http://en.angelyeast.com/contents/1193/16721.html).

**Table 3:** Chemical composition (dry weight basis (%)) of raw materials and unwashed WIS

**Chemical composition**	**Reed straw**		**Corn stover**	
	**Raw material**	**Unwashed WIS**	**Raw material**	**Unwashed WIS**
Benzene-alcohol (2:1) extractive	8.39 ± 0.10	12.14 ± 0.10	10.95 ± 0.07	19.97 ± 0.19
Glucan	40.52 ± 0.03	55.87 ± 0.03	38.75 ± 0.04	57.07 ± 0.03
Xylan	25.86 ± 0.19	3.05 ± 0.03	23.51 ± 0.18	1.80 ± 0.02
Acid-insoluble lignin	16.22 ± 0.02	17.67 ± 0.03	15.62 ± 0.16	16.89 ± 0.03
Acid-soluble lignin	2.0 ± 0.1	0.6 ± 0	2.4 ± 0	0.4 ± 0.1
Ash	3.59 ± 0.14	8.67 ± 0.10	3.65 ± 0.08	0.40 ± 0.04

### LHW pretreatment

LHW pretreatment was conducted in a 15 L digester (machine making factory of Shanxi University of Science and Technology, Shanxi, China). The digester was a cylinder, with an axis passed through its middle portion. The digester could rotate around the motor-driven axis to ensure material uniformity. The digester was electrically heated. Approximately 700 g of raw materials and 7,000 mL of deionized water were loaded in the digester. The pretreatment temperature was controlled at 210°C, the heating time to maximum temperature was 100 minutes, and pretreatment time at the maximum temperature was set to 20 minutes. The cooling down time was approximately 15 minutes. After LHW pretreatment, the WIS and the prehydrolysates were separated by filtration using a cloth bag. The WIS was divided into two fractions. In one fraction, the residual prehydrolysate was removed by a hydraulic machine and named as unwashed WIS, whereas the other fraction was washed with water until the pH reached 7 and was named as washed WIS. The unwashed and washed WIS were stored in a refrigerator at 4°C and used for the subsequent experiments. The moisture content of the unwashed WIS of reed straw and corn stover was 56.82% and 56.06%, respectively. The moisture content of the washed WIS of reed straw and corn stover was 59.39% and 62.17%, respectively. The yields of WIS after LHW pretreatment were 59.64% for reed straw and 57.34% for corn stover, respectively. The chemical compositions of unwashed WIS pretreated with LHW are also shown in Table 3.

### Tween 40 pretreatment prior to fed-batch S-SSF

Approximately 12 g of washed or unwashed WIS and Tween 40 according to a certain ratio of substrate to Tween 40 were added to 250 mL Erlenmeyer flasks. For comparison, sometimes the calcium hydroxide and sodium bisulfite of 0.5 g per 100 g oven-dried WIS were added. The pretreatment temperature was controlled at 25°C to 100°C, and the pretreatment time was set to 0 to 90 minutes. When the Tween 40 pretreatment time was 0 minutes, the substrate was directly washed using Tween 40. After the pretreatment, the mixtures were divided into two fractions with a cloth bag. The solid fraction was used for the follow-up fed-batch S-SSF. The liquid fraction was used for the Tween 40 recycle utilization experiment.

### Fed-batch S-SSF

The fermentation experiment was conducted in 100 mL Erlenmeyer flasks. A specific calculated mass of solid cellulase was first dissolved in HAc-NaAc buffer of pH 4.8 in the flask. The dosage of cellulase was 15 FPU/g to 30 FPU/g of oven-dried weight WIS, and the ratio of WIS:buffer was 1:10 (w/v). Then, the pre-weighted WIS of 1 g (on oven-dried weight) was added into the flask, and sealed with rubber stoppers equipped with syringe needles to remove the generated carbon dioxide. The flasks were placed in the water shaker. In the pre-hydrolysis phase, the medium temperature was maintained at 50°C, and the pre-hydrolysis time was fixed at 18 hours according to our previous study [27]. The initial pH in S-SSF with calcium hydroxide was 4.89 (reed straw) and 4.91 (corn stover), and with sodium bisulfite was 4.81 (reed straw) and 4.80 (corn stover), respectively. For optimization of process conditions of Tween 40 pretreatment and fed-batch S-SSF of unwashed WIS, the pre-weighted WIS of 1 g (on oven-dried weight) was fed into the Erlenmeyer flask at 6 hours of prehydrolysis time. For evaluation of different feeding methods, WIS was fed at different prehydrolysis times and fermentation times according to different fed-batch strategies given in Table 1. After the pre-hydrolysis, the medium temperature was adjusted to the fermentation temperature of 36°C and maintained during the subsequent SSF. Approximately 0.2 mL of activated yeast was added into the medium. The fermentation experiments were performed for 72 hours. The ethanol and glucose concentrations were determined using the SBA-40D Biological Sensing Analyzer (Biology Institute of the Shandong Academy of Sciences, Jinan, China). Each experiment was performed using three parallel samples and the standard error was calculated using Microsoft Excel (Redmond, WA, USA) software.

### Chemical composition analysis

The contents of xylan, acid-insoluble lignin, ash, acid-soluble lignin, and benzene-alcohol (2:1) extractives were determined according to the Chinese National Standard methods, namely, GB/T2677.9–1994, GB/T2677.8–1994, GB/T2677.3–1993, GB/T10337–1989, and GB/T2677.6–1994, respectively. The glucan content was determined according to National Renewable Energy Laboratory (NREL) methods.

The glucan content was calculated using formula (1):

(1) $$\mathrm{Glucan}\;\mathrm{content}\;\left(\%\right)=\frac{\left[\mathrm{glucose}\right]\times 0.087\times 0.9}{m}\times 100$$

where [glucose] is glucose concentration (g/L), m is mass of oven-dried solid residues (g), 0.087 is volume of acid hydrolysis liquid (L), and 0.9 is conversion factor for glucose to glucan.

The ethanol yield was calculated using formula (2):

(2) $$\mathrm{Ethanol}\;\mathrm{yield}\left(\%\right)=\frac{\left[\mathrm{EtOH}\right]}{f\times \mathrm{biomass}\times 1.111\times 0.51}\times 100$$

where [EtOH] is the ethanol concentration at the end of the fermentation minus any ethanol produced from the enzyme and medium (g/L), f is the glucan fraction of dry biomass (g/g), biomass is the dry biomass concentration at the beginning of the fermentation (g/L), 0.51 is the conversion factor for glucose to ethanol based on the stoichiometric biochemistry of yeast, and 1.111 is the conversion factor of cellulose to equivalent glucose.

## Abbreviations

CBU: Cellobiase unit; FPU: Filter paper unit; LHW: Liquid hot water; NREL: National Renewable Energy Laboratory; SSF: Simultaneous saccharification and fermentation; S-SSF: Semi-simultaneous saccharification and fermentation; WIS: Water-insoluble solids.

## Competing interests

The authors declare that they have no competing interests.

## Authors’ contributions

JL and XZL carried out the experiments, data analyses, and drafted the manuscript. RFY revised the manuscript. JZ designed the work, and participated in manuscript writing and data analysis. YBQ reviewed the paper. All authors read and approved the final manuscript.
